# Contralateral Liver Hypertrophy and Oncological Outcome Following Radioembolization with ^90^Y-Microspheres: A Systematic Review

**DOI:** 10.3390/cancers12020294

**Published:** 2020-01-27

**Authors:** Emrullah Birgin, Erik Rasbach, Steffen Seyfried, Nils Rathmann, Steffen J. Diehl, Stefan O. Schoenberg, Christoph Reissfelder, Nuh N. Rahbari

**Affiliations:** 1 Department of Surgery, Universitätsmedizin Mannheim, Medical Faculty Mannheim, Heidelberg University, 68167 Mannheim, Germany; emrullah.birgin@umm.de (E.B.); erik.rasbach@umm.de (E.R.); steffen.seyfried@umm.de (S.S.); christoph.reissfelder@umm.de (C.R.); 2 Institute of Clinical Radiology and Nuclear Medicine, Universitätsmedizin Mannheim, Medical Faculty Mannheim, Heidelberg University, 68167 Mannheim, Germany; nils.rathmann@umm.de (N.R.); steffen.diehl@umm.de (S.J.D.); stefan.schoenberg@umm.de (S.O.S.)

**Keywords:** SIRT, selective internal radiation therapy, preconditioning, hepatectomy, neoadjuvant, liver resections

## Abstract

Radioembolization with ^90^Y-microspheres has been reported to induce contralateral liver hypertrophy with simultaneous ipsilateral control of tumor growth. The aim of the present systematic review was to summarize the evidence of contralateral liver hypertrophy and oncological outcome following unilateral treatment with radioembolization. A systematic literature search using the MEDLINE, EMBASE, and Cochrane libraries for studies published between 2008 and 2020 was performed. A total of 16 studies, comprising 602 patients, were included. The median kinetic growth rate per week of the contralateral liver lobe was 0.7% and declined slightly over time. The local tumor control was 84%. Surgical resection after radioembolization was carried out in 109 out of 362 patients (30%). Although the available data suggest that radioembolization prior to major hepatectomy is safe with a promising oncological outcome, the definitive role of radioembolization requires assessment within controlled clinical trials.

## 1. Introduction

Extended liver resection for primary and secondary hepatic malignancies is dependent on an adequate future liver remnant (FLR) volume [1,2]. Preoperative measures to increase the volume of the FLR are effective in preventing posthepatectomy liver failure in marginally resectable disease, underlying liver cirrhosis, or heavily pretreated patients [3,4]. These interventions include selective occlusion of the contralateral portal venous supply via portal vein ligation or portal vein embolization (PVE) [5]. Portal vein ligation requires a laparoscopy or laparotomy with manipulation of the hepatic hilum that can render a subsequent resection more challenging, whereas PVE can be performed percutaneously with comparable hypertrophy rates [5]. Therefore, PVE has been used at most hepatobiliary units as the primary intervention to increase the FLR. However, tumor progression during the hypertrophy period after PVE has remained an unsolved issue and prevents subsequent hepatectomy in up to 40% of patients [6,7]. An alternative approach to portal vein ligation and PVE is associating liver partition and portal vein ligation for staged hepatectomy (ALPPS). Unfortunately, the results for ALPPS in patients with primary liver malignancies have been disappointing due to high perioperative morbidity and mortality rates [8].

Recently, there has been increasing interest in radioembolization with ^90^Y-microspheres (also known as selective internal radiation therapy), as this treatment offers local tumor control with simultaneous hypertrophy of the contralateral lobe [9,10]. Radioembolization (RE) is a minimally invasive procedure with transarterial delivery of ^90^Y-loaded microspheres made of glass (diameter: 25 ± 10 µm; activity per particle: 2500 Bq) or resin (diameter: 35 ± 10 µm; activity per particle: 50 Bq) [11,12]. The first reports of treatment with ^90^Y-microspheres dates back to 1965 for patients with unresectable pancreatic and liver cancer [13]. The concept of radioembolization-induced liver hypertrophy, also termed as radiation lobectomy, was first described in 2008 in patients with colorectal liver metastasis, and in 2009 in patients with hepatocellular carcinoma (HCC) [14,15]. 

Since then, several cohort studies have reported the effectiveness of RE-induced contralateral liver hypertrophy in predominantly primary liver cancer [16,17]. Moreover, a recent secondary analysis of a randomized trial revealed a higher resectability rate for patients with initially unresectable colorectal liver metastasis (CRLM) who received RE and chemotherapy compared to chemotherapy only [18]. Hence, RE seems to be a promising approach for multimodal treatment of primary and secondary liver malignancies. However, the effective hypertrophy rate and its simultaneous impact on local tumor control remains unclear. Although several reviews about ^90^Y-microspheres have been reported in the literature before, the effectiveness of ^90^Y-microspheres in liver preconditioning and its simultaneous oncological outcome for the treatment of primary and/or secondary liver malignancies have not been thoroughly assessed [10,12,16,17,19,20]. 

Therefore, the aim of this analysis was to summarize the evidence of RE as a method to induce contralateral liver hypertrophy with simultaneous ipsilateral control of tumor growth.

## 2. Results

**Figure 1:**
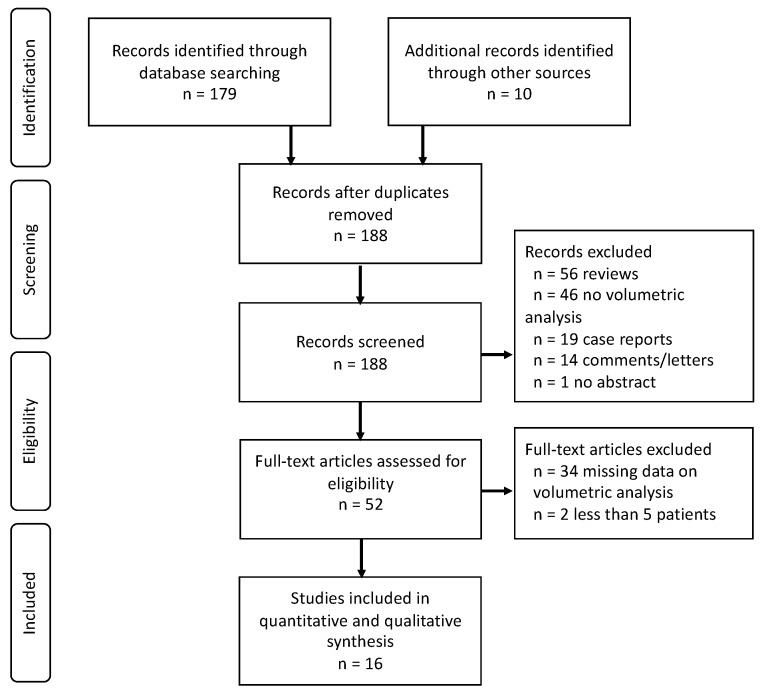
The PRISMA flow chart.

A total of 189 studies were identified by the search criteria. Following abstract and full-text screening for eligibility, 173 studies were excluded according to our inclusion criteria. Finally, 16 studies remained eligible for inclusion in this review (Figure 1).

### 2.1. Baseline Characteristics

The 16 included studies comprised a total of 602 patients who underwent RE for primary and/or secondary liver malignancies [14,15,21,22,23,24,25,26,27,28,29,30,31,32,33,34]. Patient characteristics and outcomes are summarized in Table 1. 

The median age was 68 (range: 59–79) with a predominance of men (76%). Pathological findings revealed 490 (81%) primary liver malignancies. Of these, 462 (94%) patients had hepatocellular carcinoma and 28 (6%) patients had cholangiocarcinoma. A total of 136 (22%) patients suffered from secondary liver malignancies, of which 86 (77%) patients had CRLM. Underlying liver cirrhosis was documented in 375 cases (62%). Only 8 studies (*n* = 42 patients) reported on the presence of portal hypertension (PHTN) and 9 studies (*n* = 73 patients) described the frequency of portal vein thrombosis (PVT). Prior to RE, a total of 136 patients received the following therapies: 102 patients (17%) had prior chemotherapy (CTx), 24 patients (4%) had prior local ablative therapy (LATx), and 10 patients (2%) had prior hepatectomy (Hx).

### 2.2. Contralateral Lobe Hypertrophy

The assessment of the RE-induced contralateral lobe hypertrophy is detailed in Table 2. The most commonly used modality of RE was ^90^Y-glass-microspheres (*n* = 435, 72%), whereas ^90^Y-resin-microspheres were used in 167 (28%) patients. A total of 585 (97%) patients received unilobar treatment. Bilobar treatment was conducted in 17 patients. Three studies reported on segmental RE in 15 out of 169 patients (9%) [22,25,28]. The median applied activity of yttrium was 2.1 GBq, with a median administered dose of 121 Gy. 

Only patients with unilobar treatment were assessed for measurements of contralateral liver lobe hypertrophy. The mean follow-up (F/U) was 5 months and ranged between 4 weeks and 18 months. The median kinetic growth rate (KGR) per week was 0.7% and declined continuously over time, although the maximum degree of relative contralateral hypertrophy exceeding 40% was achieved after 9 months. The ratio between future liver remnant and total liver volume (FLR/TLV) at the baseline ranged between 23.4% and 49.6%, and the FLR/TLV after RE between 27.0% and 68.6%. The KGR between the applicated microspheres showed a similar distribution: 0.8% (range: 0.3%-3%) for glass-microspheres only (*n* = 408), and 0.8% (range: 0.4–1.3%) for resin-microspheres only (*n* = 163). Only one study used both glass and resin microspheres for a total of 34 patients [21]. With regard to the underlying malignancy, the median KGR was 0.7% (range: 0.3–2.5%) in primary malignancies (*n* = 316), 0.8% (range: 0.7–1.3%) in secondary malignancies (*n* = 54), and 0.8% (range: 0.7–1.7%) in both primary and secondary malignancies (*n* = 201). Three studies assessed entirely cirrhotic patients in a cohort of HCC [21,28,32]. These patients had a similar KGR of 0.7% per week (range: 0.6–1.1%) compared to patients without liver cirrhosis with a KGR per week of 0.7% (range: 0.3–1.3%). Further subgroup analysis of patients with PVT, PHTN, previous chemotherapy, previous local ablative therapy, or previous hepatic surgery were not feasible due to heterogeneous patient cohorts and data.

### 2.3. Oncological Outcome

Details about the oncological outcome following RE were reported in 12 studies (*n* = 357 patients) and are displayed in Table 3. 

In the studies, 57 out of 360 patients (16%) developed tumor progression during a median F/U period of 11 months (range: 1–49 months). In the ipsilateral liver lobe, a total of 11 patients had progressive disease, whereas tumor progression was accounted in the contralateral liver lobe in a total of 42 patients, and 3 patients had progressive disease without further specification. Only three patients suffered from distant extrahepatic tumor progress.

Tumor response according to response evaluation criteria in solid tumors (RECIST) criteria following RE was stated in six studies (*n* = 118 patients). According to RECIST, complete and/or partial response was observed in 36 patients (31%), stable disease was noted in 65 patients (55%), and a total of 17 patients showed evidence of progressive disease (14%). Two studies (*n* = 44 patients) reported tumor response corresponding to the European Association for the Study of the Liver (EASL) and the World Health Organization (WHO) criteria [26,34]. According to WHO criteria, partial response was demonstrated in 11 patients, stable disease in 30 patients, and progressive disease in 3 patients. According to EASL criteria, complete response was depicted in 9 patients, partial response in 14 patients, and stable disease in 21 patients. Ten studies provided data on liver surgery following RE in primarily unresectable liver tumors, resulting in 109 out of 362 patients (30%) who underwent secondary surgery. The time interval of surgery after the last treatment of RE was reported in three studies with time points ranging between 2 and 30 weeks after RE [25,26,34].

### 2.4. Post-RE Treatment and Complications

Peri-interventional complications and adverse effects following RE are outlined in Table 4. 

Twelve studies disclosed RE-related adverse effects in a total of 326 patients with varying details as follows: fatigue (*n* = 78), abdominal pain (*n* = 41), hyperbilirubinemia (*n* = 22), vomiting—fever/chills without infection (*n* = 9), decreased appetite (*n* = 8), weight loss (*n* = 5), ascites (*n* = 3), encephalopathy (*n* = 1), leukopenia (*n* = 1), acute cholecystitis (*n* = 1), and pseudoaneurysm at the puncture site (*n* = 1). One study graded the adverse effects in line with the Radiation-Lobectomy Clinical Toxicity (RLCT) grade [26]. Four studies reported on postoperative complications after RE following hepatectomy according to Clavien–Dindo classification, resulting in a postoperative morbidity rate (Clavien–Dindo grade III-IVb) of 15% (*n* = 16 patients). A total of three patients (3%) died postoperatively within 90 days after surgery (Clavien–Dindo grade V) [25,26,29,34].

### 2.5. Quality Assessment

All studies were non-comparative cohort studies or clinical series, except for one matched-pair analysis [23]. The methodological quality of the included studies is summarized according to the Methodological Index for Non-Randomized Studies (MINORS) criteria in Table S1. The median MINORS score was 10 (range: 7–16). Only patients with RE were assessed for quality analysis by the MINORS scale. Thus, the control group (cohort of patients with PVE) in the study by Garlipp et al. was not considered for further MINORS score evaluation [23]. All identified studies were in retrospective fashion, except for one study [27]; however, this was non-randomized and not controlled. 

## 3. Discussion

The present review demonstrates a significant amount of contralateral lobe hypertrophy following RE with a median KGR of 0.7% per week. Up to 84% of the treated patients showed a local tumor control following RE. Finally, 30% of individuals with primarily unresectable hepatic malignancies underwent subsequent hepatic resection with decent postoperative morbidity and mortality rates in heavily pretreated patients. However, the evidence on RE-induced liver hypertrophy is primarily based on retrospective cohort studies. The impact of selection bias in these retrospective studies remains unknown and resulted in a low MINORS score. The only available prospective study was published in a small cohort of 24 patients with palliative treatment of advanced hepatocellular carcinoma [31]. Despite its prospective design, this study lacks information on the effect of RE on hypertrophy in subgroups at high risk of poor hypertrophy, such as patients with diabetes, advanced cirrhosis, and portal vein thrombosis (PVT). Although the impact of underlying liver cirrhosis on the induction of contralateral hypertrophy following RE remains controversial with comparable KGR compared to patients without liver cirrhosis, future studies need to document clinicopathologic characteristics to provide clinicians with more precise information on the regenerative potential of RE. 

The optimal FLR volume for safe hepatectomy is still uncertain. An FLR and TLV ratio of 20–30% has been suggested as a threshold for patients with normal liver parenchyma and 40–45% for patients with liver cirrhosis [35]. To date, the impact of RE-induced contralateral liver hypertrophy has been assessed in two systematic reviews, including seven and eight studies, respectively [10,17]. Teo et al. reported only the relative degree of hypertrophy regardless of the oncological outcome, and no data on the ratio of FLR to TLV. Similarly, Braat et al. assessed only the degree of contralateral liver hypertrophy irrespective of FLR/TLV, but in combination with the oncological response according to RECIST criteria. In both reviews, the small number of patients and the varying time points of volume assessments resulted in a huge range of relative degrees of liver hypertrophy between 7% to 62% and 26% to 42%, respectively. However, in the daily routine, the ratio of FLR and TLV rather than the relative degree of liver lobe hypertrophy is used to determine a safe resectability. To overcome the disadvantage of varying time points of hypertrophy assessments due to the given data in the literature, we thoroughly analyzed the KGR per week for each study in relation to the earliest F/U imaging. The KGR is a more reliable parameter than the relative degree of liver hypertrophy and might predict posthepatectomy liver failure [36]. Additionally, the KGR enables a rough prediction of the required time interval to the intended FLR/TLV threshold. In fact, kinetic studies revealed that RE induces steady hypertrophy rates over time, although there is a distinct decline of hypertrophy after 3 months [27]. In contrast to RE, PVE passes in a plateau phase after 3 weeks. [37]. Still, the best time point for a volumetric assessment following preconditioning modalities remains unclear. According to a recent randomized trial comparing the ALPPS procedure to PVE, PVE induced a mean KGR of 6 ± 5% [38]. Although the present review depicted a substantial lower KGR for RE with values ranging between 0.3% and 2.5%, certain patient selection without heavily pretreated patients might overcome these low values, and this needs to be addressed in prospective trials. In case of insufficient hypertrophy levels after RE, the option of PVE/portal vein ligation or even a combination of transarterial and transportal RE is feasible [25,39,40]. To date, the only comparative study between RE and PVE, a matched-pair analysis, demonstrated a significantly higher mean relative hypertrophy level of 62% following PVE compared to a mean relative hypertrophy level of 29% following RE [23]. However, the study design shows a strong selection and reporting bias by including two cohorts from different institutions with heavily pretreated patients in the RE group [38]. 

Despite the need for larger injection volumes and subsequent higher microembolic effects for resin-based spheres due to lower relative activity, no difference has been noted thus far in clinical response [41]. Of note, RE by microspheres does not result in macrovascular embolization; thus, the hypertrophy of the liver is probably induced by radiation effects [14,21,42]. Ultimately, the impact of dose-dependent and activity-related hypertrophy effects of ^90^Y-microspheres, as well as the role of segmental RE, remains unclear due to the lack of provided data. 

RE is a well-tolerated and safe intervention to treat patients with liver malignancies [43]. Certain clinical conditions such as PVT and/or PHTN with impaired liver function limit the application of transarterial chemoembolization but not the use of RE [44]. In comparison to transarterial chemoembolization, a postembolization syndrome or RE-induced liver disease is a rare event [12,45]. As revealed by our results, not all included studies reported on adverse events. In literature, the incidence of RE-induced liver disease (REILD) is up to 10% [46]. However, there is currently no unifying definition of REILD and the reporting of adverse effects is highly variable [47]. In our analysis, RE-related hepatotoxicity was below 10%.

According to the RECIST criteria, radiologic tumor response (partial and complete response) following RE displayed promising effects with response rates up to 96% in liver malignancies [23]. Recently, a randomized-controlled trial reported delayed disease progression in the liver following the addition of RE to standard chemotherapy in unresectable CRLM [48]. This might potentially increase resectability if subsequent liver surgery was planned [18]. To date, only a few retrospective clinical series with subsequent hepatic resections following RE are available in the literature, but with favorable oncological and surgical results. [25,49,50]. A time interval of 2 to 3 months between the last RE application and hepatic resection is recommended as optimal timing for resection [51]. However, there is also evidence for feasible secondary liver resection even 20 months following RE (*n* = 12) with acceptable morbidity (50%) and 90-day mortality (8%) rates [52]. Unfortunately, long-term results following RE and secondary liver resection are limited in the literature, but survival rates of up to 86% at 3 years and recurrence-free survival of 34 months in HCC seems to be promising for future trials [34]. Nevertheless, the only prospective pilot study of RE application with subsequent surgery revealed progressive liver and extrahepatic disease following RE in 3 out of 30 patients with subsequent contraindication for surgery [53]. 

The present review revealed several limitations for the findings of the included studies. There was a high heterogeneity with regard to RE modality, follow-up period, underlying pathology, and prior treatment before RE application across the studies. The measurements of contralateral liver lobe hypertrophy were performed at different time points, resulting in a certain effect imprecision, although the KGR was calculated to provide more robust data. Further, RE-induced adverse events were stated in 75% of the included studies, but with highly variable reporting. This might have resulted in an underestimation of adverse events following RE.

## 4. Materials and Methods 

A systematic literature research was conducted according to the PRISMA guidelines and a defined study protocol [54].

### 4.1. Search Strategy

The MEDLINE (via PubMed), EMBASE, and Cochrane Library databases were searched for studies published between 1 January 2008 and 3 January 2020. The search was limited to studies published after 2008, as the concept of radioembolization-induced liver hypertrophy was first described in 2008 [14]. A combination of the following terms as free text words and/or medical subject headings was used as search strategy: “TARE”[All Fields] OR “Yttrium 90”[All Fields] OR “selective internal radiation therapy”[All Fields] OR “radioembolisation”[All Fields] OR “radioembolization”[All Fields] AND (“hypertrophy”[MeSH Terms] OR “hypertrophy”[All Fields] OR “volumetric”[All Fields] OR “liver remnant”[All Fields] OR “liver resection”[All Fields] OR “lobectomy”[All Fields] OR “hepatectomy”[All Fields]) AND (“2008/01/01”[PDAT]: “2020/01/03”[PDAT]).

Titles and abstracts of retrieved studies were screened by two reviewers (E.B., E.R.), and full texts were obtained for potentially relevant studies. Reference lists of relevant studies were crosschecked for additional studies.

### 4.2. Eligibility Criteria

All studies that reported on quantitative contralateral liver hypertrophy following radioembolization in patients with primary or secondary liver malignancies were included. We excluded studies without data on volumetric changes of the contralateral liver lobe, reports with less than five patients, and review articles.

### 4.3. Data Collection and Definitions

Data extraction was carried out by two reviewers (E.B., E.R.). The following data were extracted: author, publication date, study design, total number of patients, age, sex, modality of radioembolization, radioembolization applicated liver site, dose, activity, time points of follow-up imaging, modality of follow-up imaging, hypertrophic volumetric change, tumor response following radioembolization, radioembolization-related adverse effects, pathology, underlying liver disease, prior treatment to radioembolization, and treatment following radioembolization and postoperative complications.

The FLR was defined as the volume of the contralateral liver lobe divided by the TLR. The degree of relative hypertrophy was defined as the increase of future liver remnant volume following RE compared to the volume of the FLR at the baseline. The primary outcome was the KGR per week assessed by the means of the relative increase FLR volume. If the KGR was not provided in the studies, the following formula was used to calculate the KGR:

$$\mathrm{KGR}=\;\frac{\left(\frac{\mathrm{FLR}}{\mathrm{TLV}}\right)\mathrm{after}\;\mathrm{treatment}-\;\left(\frac{\mathrm{FLR}}{\mathrm{TLV}}\right)\mathrm{baseline}}{\mathrm{time}\;\left(\mathrm{weeks}\;\mathrm{elapsed}\;\mathrm{between}\;\mathrm{baseline}\;\mathrm{and}\;\mathrm{treatment}\right)}$$

The following data were recorded for each study: kind of malignancy; underlying liver disease; presence of PHTN or PVT; tumor response (according to RECIST, EASL, and/or WHO criteria) [55,56]; post-radioembolization complications; and pre- and post-radioembolization treatments such as chemotherapy, radiation therapy, local ablative therapy (LATx), portal vein embolization (PVE), surgery, and postoperative morbidity. In addition, details of the performed surgical procedure were documented.

#### Risk-of-Bias Assessment

The Methodological Index for Non-Randomized Studies (MINORS) was applied to assess the quality of selected studies by two independent reviewers (E.B., E.R.) [57]. 

### 4.4. Statistical Analysis

The KGR and the relative rate of hypertrophy was presented as reported in the original articles or calculated as described above. A pooling of data was performed, and variables were presented as median and range. SPSS software version 24 was used for data tabulation.

## 5. Conclusions

In summary, the available literature shows a considerable degree of RE-induced contralateral lobe hypertrophy with favorable tumor control and even secondary surgery following RE for hepatic malignancies. However, the definitive role of RE in the treatment algorithm of liver tumors remains unclear due to missing randomized controlled trials comparing RE with standard therapies. Therefore, the promising potential of RE as an additional strategy in the multimodal treatment of liver malignancies needs to be investigated in prospective studies, particularly, with reference to the effective KGR, oncological outcome, and RE-related adverse effects. 

## Figures and Tables

**Table 1:** Baseline characteristics of patients in included papers.

Author	Year	Pts	Age ^†^	Sex (M/F)	Pathology	Underlying Liver Disease	Prior Therapy	MINORS Score
Edeline [21]	2013	34	n/a	29:5	HCC: 34	Cirrhosis: 34 PHTN: 19 PVT: 14	None	10
Fernandez-Ros [22]	2014	83	66	61:22	HCC: 52 CCC: 4 CRLM: 13 NCRLM: 14	Cirrhosis: 44 PHTN: none PVT: 8	CTx: 29	7
Gaba [15]	2009	20	67 *^§^*	16:4	HCC: 17 CCC: 3	Cirrhosis: 17 PHTN: - PVT: 5	CTx: 16 LATx: 3 Hx: 3	9
Gabr [34]	2018	31	63	n/a	HCC: 31	Cirrhosis: 14	LATx: 1 Hx: 2	16
Garlipp [23]	2014	26	59.2 ^§^	12:14	CRLM: 12 NCRLM: 14	Cirrhosis: none PHTN/PVT: -	CTx: 26	10 (16)
Goebel [24]	2017	75	67	60:15	HCC: 75	Cirrhosis: 55 PHTN: - PVT: 8	None	9
Jakobs [14]	2008	32	n/a	15:17	CRLM: 20 Other: 12	Cirrhosis: none PHTN/PVT: -	CTx/RTx: -	10
Justinger [25]	2015	13	79	8:5	CRLM: 13	Cirrhosis: none PHTN/PVT: -	CTx: 3 PVE: 1	9
Lewandowski [26]	2016	13	62	9:4	HCC: 10 CCC: 2 CRLM: 1	Cirrhosis: 5 PHTN: none PVT: -	CTx: 1 Hx: 1	11
Orcutt [27]	2018	25	71	21:4	HCC: 17 CCC: 3 CRLM: 5	Cirrhosis: 13 PHTN: 6 PVT: none	none	11
Palard [28]	2017	73	67.9 ^§^	66:7	HCC	Cirrhosis: 73 PHTN/PVT: -	CTx: 5 LATx: 18 Hx: 4	10
Rayar [29]	2015	8	68	3:5	CCC: 8	Cirrhosis: none PHTN/PVT: -	CTx: 8 PVE: 1	10
Teo [30]	2014	17	72	12:5	HCC	Cirrhosis: 12 PHTN: 11 PVT: -	none	10
Teo [31]	2018	24	66	24:0	HCC	Cirrhosis: 16 PHTN: 6 PVT: 6	none	10
Theysohn [32]	2014	45	72 ^§^	36:9	HCC	Cirrhosis: 45 PHTN/PVT: -	-	10
Vouche [33]	2013	83	68	66:17	HCC: 67 CCC: 8 CRLM: 8	Cirrhosis: 47 PHTN: - PVT: 32	CTx: 14	7

Pts: patients, M/F: male-to-female ratio, MINORS: Methodological Index for Non-Randomized Studies, HCC: hepatocellular carcinoma, CCC: cholangiocarcinoma, CRLM: colorectal liver metastasis, NCRLM: non-colorectal liver metastasis, PHTN: portal hypertension, PVT: portal vein thrombosis, CTx: chemotherapy, PVE: portal vein embolization, RTx: radiation therapy, LATx: local ablative therapy, Hx: hepatectomy, -: data not available. ^†^ values are median; ^§^ values are mean.

**Table 2:** Overview of radioembolization (RE)-induced hypertrophy.

Author	RE Mod	RE Dose/Activity ^†^	Mean F/U Period Following RE	KGR/Week in %	FLR/TLV at Baseline in %	FLR/TLV After ^90^Y in %	Mean Degree of Relative Hypertrophy Level in %
Edeline [21]	G+R	122 Gy, 2.5 GBq	3 mo	1.1	42.3	55.5	+ 29
Fernandez-Ros [22]	R	-	4–8 wks 10–26 wks >26 wks	0.8 - -	29.0 ^§^ - -	32 ^§^ 40 ^§^ 50 ^§^	+ 12.6 + 23.0 + 39.3
Gaba [15]	G	132 Gy 2.2 GBq	18 mo	1.9	42.9	68.6	+ 40.0
Gabr [34]	G	140 Gy	2.9 mo	2.5 *	35.0 *	45.0 *	+ 23.3 (unilobar) + 9 (segmental)
Garlipp [23]	R	1.2 GBq	46 days ^†^	0.7	23.4 ^§^	27.9 ^§^	+ 29; + 25.3 †
Goebel [24]	G	113 Gy ^§^ 2.9 GBq ^§^	1 mo 3 mo 6 mo 9 mo (*n* = 61)	0.9 0.8 0.7 0.5	33.9 - - -	37.6 44.2 50.9 53.1	+ 16.9 + 29.3 + 40.9 + 43.4
Jakobs [14]	G	120 Gy	4 wks	0.6 *	31.2 *	44.4 *	+ 21.2; + 8.7 †
Justinger [25]	R	1.3 GBq	8 wks	1.3	30.4 ^§^	40.4 ^§^	-
Lewandowski [26]	G	154 Gy	40 days ^†^	1.7	33.0	43.9	+ 30
Orcutt [27]	G	132 Gy	1 mo; 1.1 mo ^†^ 3 mo; 3.8 mo ^†^ 6 mo; 6.3 mo ^†^	0.8 0.5 0.4	33.0 - -	37.5 41.9 44.5	+ 16.7; + 11 ^†^ + 31.1; + 17.4 ^†^ + 38.5; + 31.3 ^†^
Palard [28]	G	-	5.9	0.7	49.6 ^§^	66.9 ^§^	+ 35.4
Rayar [29]	G	2 GBq	7.6 mo ^†^	0.3	27.2	35.9	+ 8.7
Teo [30]	R	-	5.7 mo ^†^	0.5	32.7 ^§^	45.5 ^§^	+ 34.2
Teo [31]	R		4–6 wks 8–12 wks (*n* = 22)	0.3 -	31.7 ^§^ -	33.8 ^§^ 39.7 ^§^	+ 5.6; + 3 ^†^ + 21.2; + 9 ^†^
Theysohn [32]	G	-	1 mo 3 mo 6 mo 9 mo 12 mo	0.7 0.8 0.6 0.5 0.4	35.0 ^§^ - - - -	37.7 45.4 50.4 ^§^ 54.0 56.5	+ 7.1 + 22.9 + 30.8 + 35.7 + 40.1
Vouche [33]	G	112 Gy	1 mo (*n* = 80) 45 days to 3 mo (*n* = 34) 3-6 mo (*n* = 42) 6-9 mo (*n* = 28) >9 mo (*n* = 25)	0.6 - - - -	24.4 - - - -	27.0 27.8 34.7 34.3 36.4	+ 7 † + 24 † + 35 † + 36 † + 45 †

RE: radioembolization, mod: modality, F/U: follow-up, KGR: kinetic growth rate, FLR: future liver remnant, TLV: total liver volume, ^90^Y: treatment with yttrium, G: glass microspheres, R: resin microspheres, mo: months, wks: weeks, -: data not available. ^†^ values are median, ^§^ values are mean, * only patients with unilobar treatment were considered.

**Table 3:** Overview of the oncological outcome.

Author	Median F/U Period	Tumor Location (Liver Site)	Tumor Progress and Location	Tumor Response (RECIST)	Post-RE Surgery
Edeline [21]	2 mo	Right: 23 Left: 11	Ipsilateral: 3	PR: 7 SD: 17 PD: 3	-
Fernandez-Ros [22]	-	Right: 66 Left: 17	-	-	Hx+LTx: 15
Gaba [15]	49 mo	Right: 20	None	PR: 14 SD: 6	Hx: 1 LTx: 1
Gabr [34]	1 mo	Right: 25 Left: 6	Location not specified: 3	CR: 9 ^#^; 0 * PR: 9 ^#^; 8 * SD: 13 ^#^; 20 * PD: 0 ^#^; 3 *	Hx: 31
Garlipp [23]	3 mo	Right: 26	Contralateral: 1	CR: 1 PR: 5 SD: 19 PD: 1	-
Goebel [24]	-	Right: 75	-	-	-
Jakobs [14]	-	Bilobar: 32	-	-	-
Justinger [25]	5.1 mo	Right: 10 Left: 1 Bilobar: 2	Contralateral: 1 Extrahepatic: 1	-	Hx: 11
Lewandowski [26]	6.3 mo	Right: 13	None	CR: 1 ^#^; 0 * PR: 5 ^#^; 3 * SD: 8 ^#^; 10 *	Hx: 13
Orcutt [27]	6 mo	Right: 25	None	-	-
Palard [28]	12.5 mo	-	Ipsilateral: 8 Contralateral: 14	-	Hx: 9
Rayar [29]	16 mo	Right: 3 Bilobar: 5	None	-	Hx: 8
Teo [30]	6 mo	Right: 17	Contralateral: 4	CR: 2 PR: 5 SD: 6 PD: 4	Hx: 1
Teo [31]	12 mo	Right: 22Left: 2	Contralateral: 5	PR: 2 SD: 17 PD: 5	Hx: 8
Theysohn [32]	-	Right: 45	-	-	-
Vouche [33]	9 mo	Right: 83	Contralateral: 17	Missing: 79 PD: 4	Hx: 5 LTx: 6
Total	11 mo	Right: 453 Left: 37 Bilobar: 39	Ipsilateral: 11/360 (3%) Contralateral: 42/360 (12%) Extrahepatic: 1/360 (1%) Not specified: 3/360 (1%)	CR: 3/118 (3%) PR: 33/118 (28%) SD: 65/118 (55%) PD: 17/118 (14%)	109/362 (30%)

F/U: follow-up, RE: radioembolization, RECIST: response evaluation criteria in solid tumors, mo: months, Hx: hepatectomy, LTx: liver transplantation, CR: complete response, PR: partial response, SD: stable disease, PD: progressive disease, -: data not available, * according to World Health Organization (WHO) criteria, ^#^ according to European Association for the Study of the Liver (EASL) response criteria.

**Table 4:** Overview of complications and adverse effects following radioembolization.

Author	RE-Related Hepatotoxicity	Other RE-Related AE	RE-Related Mortality
Edeline [21]	Hyperbilirubinemia: 6 Ascites: 3 Encephalopathy: 1	none	2
Fernandez-Ros [22]	-	-	-
Gaba [15]	Hyperbilirubinemia: 9	none	none
Gabr [34]	Hyperbilirubinemia: 6	none	none
Garlipp [23]	none	Acute cholecystitis: 1 Leukopenia: 1	none
Goebel [24]	-	-	-
Jakobs [14]	none	none	none
Justinger [25]	none	none	none
Lewandowski [26]	none	Fatigue: 10 Abdominal pain: 7 Vomiting: 4	none
Orcutt [27]	none	Fatigue: 17 Abdominal pain: 15 Vomiting: 8 Appetite loss: 8 Diarrhea: 2	none
Palard [28]	-	-	-
Rayar [29]	none	none	none
Teo [30]	none	none	none
Teo [31]	none	none	2
Theysohn [32]	-	-	-
Vouche [33]	Hyperbilirubinemia: 1	Fatigue: 51 Abdominal pain: 19 Fever: 9 Vomiting: 6 Weight loss: 5 Diarrhea:1 Pseudoaneurysm: 1	none
Total	26/326 (8%) Hyperbilirubinemia: 22 Ascites: 3 Encephalopathy: 1	165/326 (50%) Fatigue: 78 Abdominal pain: 41 Vomiting: 18 Fever: 9 Appetite loss: 8 Weight loss: 5 Other: 6	4/326 (1%)

RE: radioembolization, AE: adverse effects, -: data not available.

## Supplementary Materials

The following are available online at https://www.mdpi.com/2072-6694/12/2/294/s1: Table S1: Quality assessment of studies according to MINORS score.
