# Supplementary material for: Contralateral Liver Hypertrophy and Oncological Outcome Following Radioembolization with 90Y-Microspheres: A Systematic Review
Source: Cancers (Basel). 2020 Jan 27;12(2):294. doi: 10.3390/cancers12020294 (PMC7072354; doi:10.3390/cancers12020294)
Supplement: Supplementary file 1 [file cancers-12-00294-s001.pdf]

Table S1. Quality assessment of studies according to MINORS score.

Author and year, study design	Aim of the study	Inclusion of consecutive patients	Prospective collection of data	Endpoint appropriate to the study aim	Unbiased evaluation of endpoints	F/U period appropriate to the major endpoint	Loss to F/U not exceeding 5%	Sample calculation	Score
Edeline 2013, retrospective cohort study [21]	2<br><br>Evaluation of RE-induced liver volume changes on consecutive HCC with underlying cirrhosis<br>(n = 26)	2<br><br>Definitive inclusion criteria were present	0<br><br>Not reported	2<br><br>Volume measurements as absolute (median, IQR) and relative increase (mean, 95%CI)	0<br><br>Operator bias: single operator<br><br>Outcome bias (no information about volumetric assessment)<br><br>Selection bias	2<br><br>Evaluation at approximately 3 months seems appropriate	2<br><br>All patients were evaluated	0<br><br>Not reported	10/16
Fernandez-Ros 2014, retrospective cohort study [22]	2<br><br>Evaluation of RE-induced volume effects on the liver and spleen in primary and secondary liver malignancies<br>(n = 83)	2<br><br>Definitive inclusion criteria were present	0<br><br>Not reported	2<br><br>Volume measurement as absolute (mean, SD) and relative increase (mean, SD)<br><br>Number of resected patients available	0<br><br>Operator bias: two radiologists<br><br>Outcome bias (no information about volumetric assessment)<br><br>Selection bias	1<br><br>Time intervals are inconsistent: 4–8, 10–26 and >26	0<br><br>Unknown number of patients lost to F/U	0<br><br>Not reported	7/16
Gaba 2009, retrospective cohort study [15]	2<br><br>Evaluation of RE-induced volume effects on the liver in primary liver malignancies<br>(n = 20)	2<br><br>Definitive inclusion criteria were present	0<br><br>Not reported	2<br><br>Volume measurements as absolute (median, 95%CI, range) and relative increase (median, 95%CI)<br><br>Number of resected patients available	0<br><br>Operator bias (no information about operator)<br><br>Outcome bias: Technique of image assessment was reported<br><br>Selection bias	1<br><br>Time intervals are extreme: 2–49 months	2<br><br>All patients were evaluated	0<br><br>Not reported	9/16
Gabr 2018, Retrospective cohort study [34]	2<br><br>Evaluation of short- and long-term outcomes of	2<br><br>Definitive inclusion	0<br><br>Not reported	2<br><br>Outcome of resected patients available	0<br><br>Operator bias (no information about operator)	2<br><br>Evaluation at approximately	2<br><br>All patients were evaluated	0<br><br>Not reported	10/16

	patients with HCC who underwent hepatic resection following RE	criteria were present		Secondary Endpoint: Volume measurement as relative increase (median, IQR)	Outcome bias:<br>No information of excluded patients (n = 91)<br><br>Selection bias	3 months seems appropriate			
Garlipp 2014, retrospective matched-pair analysis [23]	2<br><br>Comparison between PVE-induced and RE-induced volume effects on the contralateral liver lobe in secondary liver malignancies (n = 26 vs. n = 26)	2<br><br>Definitive inclusion criteria were present	0<br><br>Not reported	2<br><br>Volume measurements as absolute (mean, median, SD) and relative increase (mean, median, SD)	0<br><br>Operator bias (no information about operator)<br><br>Outcome bias:<br>Technique of image assessment was reported<br><br>Selection bias	2<br><br>Evaluation at 6 weeks after SIRT seems appropriate	2<br><br>All patients were evaluated	0<br><br>Not reported	10/16 (16/24) *
Goebel 2017, Retrospective cohort study [24]	2<br><br>Evaluation of RE-induced volume effects on the liver and predictive parameters for contralateral liver lobe hypertrophy in HCC (n = 75)	2<br><br>Definitive inclusion criteria were present	0<br><br>Not reported	2<br><br>Volume measurements as absolute (mean, SD, range) and relative increase (mean, SD, range)	0<br><br>Operator bias: one experienced radiologist<br><br>Outcome bias:<br>Technique of image assessment was reported<br><br>Selection bias	2<br><br>Evaluation at 1, 3 and 6 months after SIRT seems appropriate	1<br><br>n = 14 patients were lost to F/U after 6 months.	0<br><br>Not reported	9/16
Jakobs 2008, Retrospective cohort study [14]	2<br><br>Evaluation of RE-induced morphologic changes (namely fibrosis, portal hypertension and volume effects) in secondary liver malignancies (n = 32)	2<br><br>Definitive inclusion criteria were present	0<br><br>Not reported	2<br><br>Volume measurements as relative (mean, median) increase	0<br><br>Operator bias: two radiologists with different experience<br><br>Outcome bias: Technique of image assessment was reported<br><br>Selection bias	2<br><br>Evaluation at 4 weeks seems appropriate	2<br><br>All patients were evaluated	0<br><br>Not reported	10/16
Justinger 2015,	2	0	1	2	0	2	2	0<br><br>Not reported	9/16

Retrospective clinical series [25]	Evaluation of RE-induced secondary surgery in marginally resectable secondary liver malignancies (n = 13)	No further specified inclusion criteria	Prospective database with 127 items (no further details)	Number of resected patients available<br><br>Secondary Endpoint: Volume measurements as relative (mean, SD) increase	Outcome/Operator bias (no information of technique of image assessment or operator)<br><br>Selection/Reporting bias due to clinical series	Evaluation of liver volume at 2 months and median F/U 26 months (1–54 months) seems appropriate	All patients were evaluated		
Lewandowski 2016, Retrospective cohort study [26]	2<br><br>Evaluation of RE-induced secondary surgery in marginally resectable primary and secondary liver malignancies (n = 13)	2<br><br>Definitive inclusion criteria were present	1<br><br>Prospective database (no further details)	2<br><br>Number of resected patients available<br><br>Secondary Endpoint: Volume measurement as relative increase (median, range)	0<br><br>Operator bias (no information about operator)<br><br>Outcome bias: Technique of image assessment was reported<br><br>Reporting bias (no information about the database)<br><br>Selection bias	2<br><br>Median F/U 40 days (23–190) seems appropriate for primary endpoint, however, heterogeneous and missing exact time points for evaluation of liver volume	2<br><br>All patients were evaluated	0<br><br>Not reported	11/16
Orcutt 2018, Retrospective cohort study [27]	2<br><br>Evaluation of RE-induced volume effects<br>In primary and secondary liver malignancies and assessment of a model to predict contralateral hypertrophy (n = 25)	2<br><br>Definitive inclusion criteria were present	1<br><br>Prospective database (no further details)	2<br><br>Volume measurements as absolute (median, range) and relative (mean, median, range) increase	0<br><br>Operator bias: one surgical oncologist<br><br>Outcome bias: Technique of image assessment was reported<br><br>Reporting bias (no information about the database)<br><br>Selection bias	2<br><br>Evaluation at 1, 3 and 6 months after SIRT seems appropriate	2<br><br>All patients were evaluated	0<br><br>Not reported	11/16
Palard 2017, Retrospective cohort study [28]	2<br><br>Evaluation of RE-induced	2<br><br>Definitive inclusion	0<br><br>Not reported	2<br><br>Volume measurements as	0<br><br>Operator bias (no information about operator)	2<br><br>Evaluation at 4–8 weeks, then	2<br><br>All patients were evaluated	0<br><br>Not reported	10/16

	contralateral hypertrophy and its association with dosimetric parameters<br>(n = 73)	criteria were present		absolute (mean, SD) and relative (mean, SD) increase<br><br>Number of resected patients available	Outcome bias: Technique of image assessment was reported<br><br>Selection bias	every 12–16 weeks (mean 5.9 ± 3.4 months) after SIRT seems appropriate			
Rayar 2015, Retrospective cohort study [29]	2<br><br>Evaluation of systematic chemotherapy-induced and RE-induced secondary surgery in marginally resectable CCC<br>(n = 8)	2<br><br>Definitive inclusion criteria were present	0<br><br>Not reported	2<br><br>Number of resected patients available<br><br>Secondary Endpoint: Volume measurement as absolute (median, range) and relative (mean) increase	0<br><br>Operator bias (no information about operator)<br><br>Outcome bias: Technique of image assessment was reported<br><br>Selection bias	2<br><br>Median F/U 15.6 months (4–40.7) after CTx and 7.2 months (0.13–36.4) after surgery seems appropriate for primary endpoint, however, heterogeneous and missing exact time points for evaluation of liver volume	2<br><br>All patients were evaluated	0<br><br>Not reported	10/16
Teo 2014, Retrospective cohort study [30]	2<br><br>Evaluation of RE-induced liver volume changes in HCC and impact of underlying liver disease on hypertrophy<br>(n = 17)	2<br><br>Definitive inclusion criteria were present	0<br><br>Not reported	2<br><br>Volume measurements as absolute (mean, SD) and relative (mean, SD) increase<br><br>Subgroup analysis of liver hypertrophy based on underlying liver disease<br><br>Number of resected patients available	0<br><br>Operator bias: one senior radiologist<br><br>Outcome bias: Technique of image assessment was reported<br><br>Selection bias	2<br><br>Mean F/U of 5.7 months (2–12 months) seems appropriate	2<br><br>All patients were evaluated	0<br><br>Not reported	10/16
Teo 2018, Prospective cohort study	2	2	2	2	0	2	0	0<br><br>Not reported	10/16

[31]	Evaluation of early RE-induced contralateral liver volume changes in HCC<br>(n = 24)	Definitive inclusion criteria were present	Data was prospectively recorded	Volume measurements as absolute (mean, SD, median, range) and relative (mean, SD, median, range) increase<br><br>Number of resected patients available	Operator bias: one senior radiologist<br><br>Outcome bias: Technique of image assessment was reported	Evaluation at 4–6 weeks and 8-12 weeks after SIRT seems appropriate	n = 2 patients lost to F/U at 8–12 weeks		
Theysohn 2014, Retrospective cohort study [32]	2<br><br>Evaluation of RE-induced liver volume changes in HCC with underlying cirrhosis<br>(n = 45)	2<br><br>Definitive inclusion criteria were present	0<br><br>Not reported	2<br><br>Volume measurements as absolute (mean, 95%CI) increase and relative increase (mean)	0<br><br>Operator bias: one experienced radiologist<br><br>Outcome bias: Technique of image assessment was reported<br><br>Selection bias	2<br><br>Evaluation at 1, 3, 6, 9 and 12 months after SIRT seems appropriate	2<br><br>All patients were evaluated	0<br><br>Not reported	10/16
Vouche 2013, Retrospective cohort study [33]	2<br><br>Evaluation of RE-induced liver volume changes in primary and secondary liver malignancies<br>(n = 83)	2<br><br>Definitive inclusion criteria were present	0<br><br>Not reported	2<br><br>Volume measurements as absolute (median, range) and relative (median, range) increase	0<br><br>Operator bias (no information about operator)<br><br>Outcome bias: Technique of image assessment was reported<br><br>Selection bias	1<br><br>Evaluation at 3-5 weeks, 6–12 weeks, 12–24 weeks, 24–36 weeks, >36 weeks seems appropriate, however, there is a substantial number of patients lost to F/U	0<br><br>Various number of patients lost to F/U at each time point:<br>n = 3 at 3–5 weeks,<br>n = 49 at 6–12 weeks,<br>n = 41 at 12–24 weeks, n = 55 at 24–36 weeks, n = 58 at >36 weeks	0<br><br>Not reported	7/16

* Additional criteria for Garlipp et al. (adequate control group (gold standard); contemporary groups (same time interval) – baseline equivalence of groups, adequate statistics).

Author and year, study design	adequate control group	contemporary groups	Baseline equivalence of groups	Adequate statistics

Garlipp 2014, retrospective matched-pair analysis [23]	2<br>PVE is the gold standard	0<br>Historic comparison: PVE between 1987-2005 RE between 2006–2010	2<br>Matched-pair	2<br>ANOVA
